# NT-proBNP correlates with LVEF decline in HER2-positive breast cancer patients treated with trastuzumab

**DOI:** 10.1186/s40959-019-0039-4

**Published:** 2019-05-28

**Authors:** Nathalie I. Bouwer, Crista Liesting, Marcel J. M. Kofflard, Sylvia M. Sprangers-van Campen, Jasper J. Brugts, Jos J. E. M. Kitzen, Michael A. Fouraux, Mark-David Levin, Eric Boersma

**Affiliations:** 1 Department of Internal Medicine, Albert Schweitzer Hospital, 3300 AK, Dordrecht, South-Holland The Netherlands; 2 Department of Cardiology, Albert Schweitzer Hospital, 3300 AK, Dordrecht, South-Holland The Netherlands; 3 Department of Cardiology, Erasmus MC, University Medical Center Rotterdam, 3000 CA, Rotterdam, South-Holland The Netherlands; 4 Result Laboratorium C.V, Albert Schweitzer Hospital, 3300 AK, Dordrecht, South-Holland The Netherlands

**Keywords:** Cardiotoxicity, HER2-positive breast cancer, NT-proBNP, Trastuzumab, LVEF decline

## Abstract

**Background:**

Early identification of cardiac dysfunction by non-invasive imaging in HER2-positive breast cancer patients treated with trastuzumab is challenging. In particular multigated acquisition (MUGA) scan, which is most widely used, is unable to detect subclinical cardiac changes. The use of N-terminal pro-brain natriuretic peptide (NT-proBNP), a serum biomarker of myocardial stress, might improve timely diagnosis.

**Methods:**

This prospective, single-center, cohort study included patients with HER2-positive breast cancer who started trastuzumab therapy. Echocardiography was scheduled at regular intervals every 3 months during one year follow-up for cardiac function monitoring. For research purposes, NT-proBNP was determined at the same time points. Trastuzumab-induced cardiotoxicity (TIC) was the primary study endpoint, defined as a left ventricular ejection fraction (LVEF) < 45%, and/or an absolute decline in LVEF > 10% since inclusion, and/or the incidence of a clinical cardiac event.

**Results:**

A total of 135 patients were enrolled between April 2008 and June 2016, with a median age of 54 years (IQR: 47–61). By three-dimensional echocardiography (3DE), the median LVEF at baseline was 62% (IQR: 58–65). At a median of 6 months (IQR: 5–11), 45 patients (33%) reached the study endpoint of TIC. Patients with TIC had a mean change of − 9.5% in LVEF (95% CI -7.2 to − 11.7; *p* = 0.001) during 1 year of trastuzumab treatment. Both NT-proBNP at baseline (HR 1.04, 95% CI 1.02–1.07; *p* = 0.003) and LVEF decline during anthracycline treatment prior to the start of trastuzumab (HR 1.16, 95% CI 1.07–1.25; *p* < 0.001) were independently associated with development of TIC. The level of NT-proBNP during follow-up was associated too with development of TIC (HR 1.06 per 10 pmol/l difference, 95% CI 1.02–1.10; *p* = 0.008). No steadily or sudden increase in NT-proBNP prior to TIC was observed.

**Conclusions:**

NT-proBNP cannot be used as a surrogate monitoring tool for trastuzumab-induced cardiotoxicity in HER2-positive breast cancer patients during the first year of treatment. Patients showing an LVEF decline during anthracycline pre-treatment appeared vulnerable for trastuzumab-induced cardiotoxicity.

**Electronic supplementary material:**

The online version of this article (10.1186/s40959-019-0039-4) contains supplementary material, which is available to authorized users.

## Introduction

The identification of cardiac dysfunction in Human Epidermal growth Receptor 2 (HER2) positive breast cancer patients treated with trastuzumab is challenging, but crucial in order to prevent the development of heart failure in these patients. Trastuzumab (Herceptin®, Genetech, San Francisco CA) is a highly-effective anti-cancer drug that is widely used in patients with HER2-positive breast cancer. Addition of trastuzumab to (neo)adjuvant chemotherapy in patients with HER2-positive breast cancer improved disease-free survival (DFS) and overall survival (OS) impressively. [1–3] However, trastuzumab may cause cardiotoxicity, foremost an impairment of the left ventricular ejection fraction (LVEF), which may adversely affect the prognosis and limit quality of life.

In clinical practice, it is essential to identify early subclinical cardiac dysfunction in breast cancer patients treated with trastuzumab. Clinical symptomatic heart failure might than be prevented by timely prescription of cardio-protective medication, or by interruption or even discontinuation of trastuzumab treatment, because trastuzumab-induced cardiotoxicity is (partially) reversible. [4] Multigated acquisition (MUGA) scans are widely used to monitor cardiac function in this patient population, but identification of cardiotoxicity by this technique is challenging. MUGA scans not only provide LVEF assessments with high inter- and intra-observer variability [5], but also fail to detect early subclinical cardiac alterations, because of initial compensatory mechanisms of the left ventricle to prevent functional cardiac impairment. [6] In addition, cardiac monitoring by serial MUGA scans will pose a high radiation burden to the patient. [7] Echocardiography, another frequently used cardiac monitoring approach, overcomes certain limitations of MUGA scans as echocardiography evaluates the complete cardiac structure and lacks radiation exposure.

Therefore, more advanced and sensitive diagnostic strategies are needed, and cardiac biomarkers, including N-terminal pro-brain natriuretic peptide (NT-proBNP) might be useful in this respect.

NT-proBNP is a peptide stored in, and secreted predominantly from, membrane granules in the ventricles of the heart in response to increased intra cardiac pressure. [8] NT-proBNP is an established serum biomarker for the diagnosis of heart failure [9], and is associated with adverse prognosis in heart failure patients. As trastuzumab-based therapy may induce ventricle wall stress, small changes in NT-proBNP levels can potentially be detected prior to an LVEF decline.

Studies investigating the association between NT-proBNP levels and cardiotoxicity in breast cancer patients showed inconclusive results. [10–17] Two large prospective studies demonstrated increased NT-proBNP levels in breast cancer patients with cardiotoxicity [10, 11], but several others failed. [12, 13, 15–17] These studies are hampered by a low incidence of LVEF declines compared with population based, retrospective studies (9% vs. 19%) [11, 18, 19] or predominant focus on anthracyclines instead of trastuzumab. [10]

The current, prospective cohort study was designed to overcome these limitations. We aimed to assess the potency of a screening-strategy utilizing repeatedly measured NT-proBNP levels to detect trastuzumab-induced cardiotoxicity measured with three-dimensional echocardiography (3DE) in a representative cohort of HER2-positive breast cancer patients.

## Methods

### Selection and description of participants

This prospective cohort study included women with HER2-positive breast cancer, who started trastuzumab therapy between April 2008 and June 2016 in the Albert Schweitzer Hospital (ASZ), a large teaching hospital in Dordrecht, the Netherlands.

Patients were excluded from the study in case of baseline LVEF < 45%, presence of cardiac dysfunction, ischemic heart disease, valvular heart disease, severe renal dysfunction or hepatic dysfunction or known intolerability for trastuzumab therapy. This study was approved by the institutional review board of the ASZ and was conducted according to the Declaration of Helsinki. All participants provided written informed consent.

### Procedures

Indications for trastuzumab therapy included (neo-) adjuvant (early-stage) and metastatic (advanced-stage) breast cancer with overexpression of the HER2-receptor. Patients were treated according to prevailing guidelines. [20] In patients with early-stage breast cancer, trastuzumab was preceded by 4 courses of doxorubicin 50–60 mg/m^2^ and cyclophosphamide 500–600 mg/m^2^ once every 3 weeks. After these 4 courses, trastuzumab 2–4 mg/kg was administered in combination with paclitaxel 80 mg/m^2^ weekly for 12 cycles, followed by mono-therapy trastuzumab 6 mg/kg every three weeks for one year. In patients with advanced-stage breast cancer, 6 courses of trastuzumab 2–4 mg/kg in combination with paclitaxel 80 mg/m^2^ were administered as initial treatment, after which trastuzumab 6 mg/kg was continued once every three weeks until relapse of breast cancer or until the development of cardiotoxicity or other reasons to stop trastuzumab. [21] In this study, patients were studied during the first year of trastuzumab treatment.

### Echocardiography and laboratory assessments

Echocardiography and laboratory assessments were scheduled during the first year of follow-up (Additional file 1: Figure S1). Within two weeks before or after blood collection, 3DE was systematically performed, with exclusion of eight days after the first trastuzumab administration (Additional file 1: Figure S1). Although the MUGA scan is currently most used in monitoring the cardiac function in these patients, ECG-gated triplane 3DE was used as routine cardiac monitoring because of the high accuracy, lack of radiation exposure, comprehensive evaluation of cardiac structure and reproducibility. [5] No MUGA scans were performed during the year of follow-up.

The 3DE images were obtained using a 1.5 to 3.6 MHz 3V probe with the Vivid 7 echo system (GE Vingmed Ultrasound, Trondheim, Norway). Gain and compression were set at 50%, and harmonic imaging was also used. Data sets were acquired from the parasternal long-axis, apical and subcostal position. Left ventricle (LV) volumes and LVEF were measured off line by means of summation of disc methodology with dedicated software (Echopac). The data set was aligned in 2 orthogonal planes along the long axis of the LV with clear depiction of the mitral valve and the LV apex. End diastolic measurements corresponded with the largest chamber size, whereas end systolic measurements corresponded with the smallest chamber size. The LVEF was determined as the difference between end diastolic volume (EDV) and end systolic volume (ESV), relative to the EDV. The echocardiograms were recorded by two independent experts at the echo core laboratory and evaluated by one cardiologist for research objectives.

Venous blood was systematically collected at the following time points: immediately before the start of anthracycline chemotherapy (in early-stage patients only), immediately before the start of trastuzumab, eight days after the start of trastuzumab and subsequently once every 12 weeks until 48 weeks after start of trastuzumab (Additional file 1: Figure S1). At each time point, the NT-proBNP level (Dimension Vista 500, Siemens Healthcare Diagnostics, Deerfield, Illinois) was measured according to the manufacturer’s instructions. Critical value for the NT-proBNP assay was < 10%. Treating physicians were not aware of the patients’ NT-proBNP levels.

### Study endpoints

The primary endpoint was the occurrence of trastuzumab-induced cardiotoxicity (TIC), which was defined as an LVEF < 45% at any scheduled follow-up time point and/or an absolute decline in LVEF of > 10% relative to the measurement at study start – these thresholds are used by the National Cancer Research Institute as definition to interrupt trastuzumab treatment and start ACE inhibitors [22] – and/or any cardiac event for which the patient was hospitalized, including atrial fibrillation, unstable angina pectoris, acute coronary syndrome, and symptomatic heart failure. Patients who died in the year of follow-up were censored at the last available 3DE date.

### Statistical analysis

Categorical baseline data are presented as numbers and percentages. Normality of continuous baseline data was evaluated by Shapiro-Wilk tests. Normal distributed data were presented as mean values ± one standard deviation (SD), and non-normal distributed data as median values and interquartile range (IQR).

Cox proportional hazard regression was used to analyze the relation between the following baseline characteristics and the development of TIC: age at diagnosis of breast cancer, LVEF at baseline, decline in LVEF during anthracycline treatment, and NT-proBNP at baseline. The decline in LVEF during anthracycline treatment was calculated as the difference between the LVEF at start trastuzumab and the LVEF at start anthracycline treatment.

Nonlinear mixed effect (NLME) models were used to study the LVEF and NT-proBNP change over time, as well as the relation between repeatedly measured NT-proBNP and LVEF. Echocardiography and blood collection was not necessarily performed at the same date. For this analysis, practically, we considered the closest NT-proBNP measurement within 30 days before or after an LVEF measurement as the corresponding value.

Joint modeling (JM) was applied to study the relation of repeatedly measured NT-proBNP with the incidence of TIC during follow-up. The JM combined an NLME model, describing the temporal evolution of NT-proBNP, with a Cox proportional hazard regression model, describing the time-to-event process.

Data analyses were performed using SPSS software, version 24.0 (SPSS, IBM, Chicago, Illinois, USA) and R statistical software (version 3.4.3), in particular the packages “nlme” and “JM”. Statistical significance of all tests was set at a two-tailed *p*-value of less than 0.05.

## Results

### Patient characteristics

**Fig. 1:**
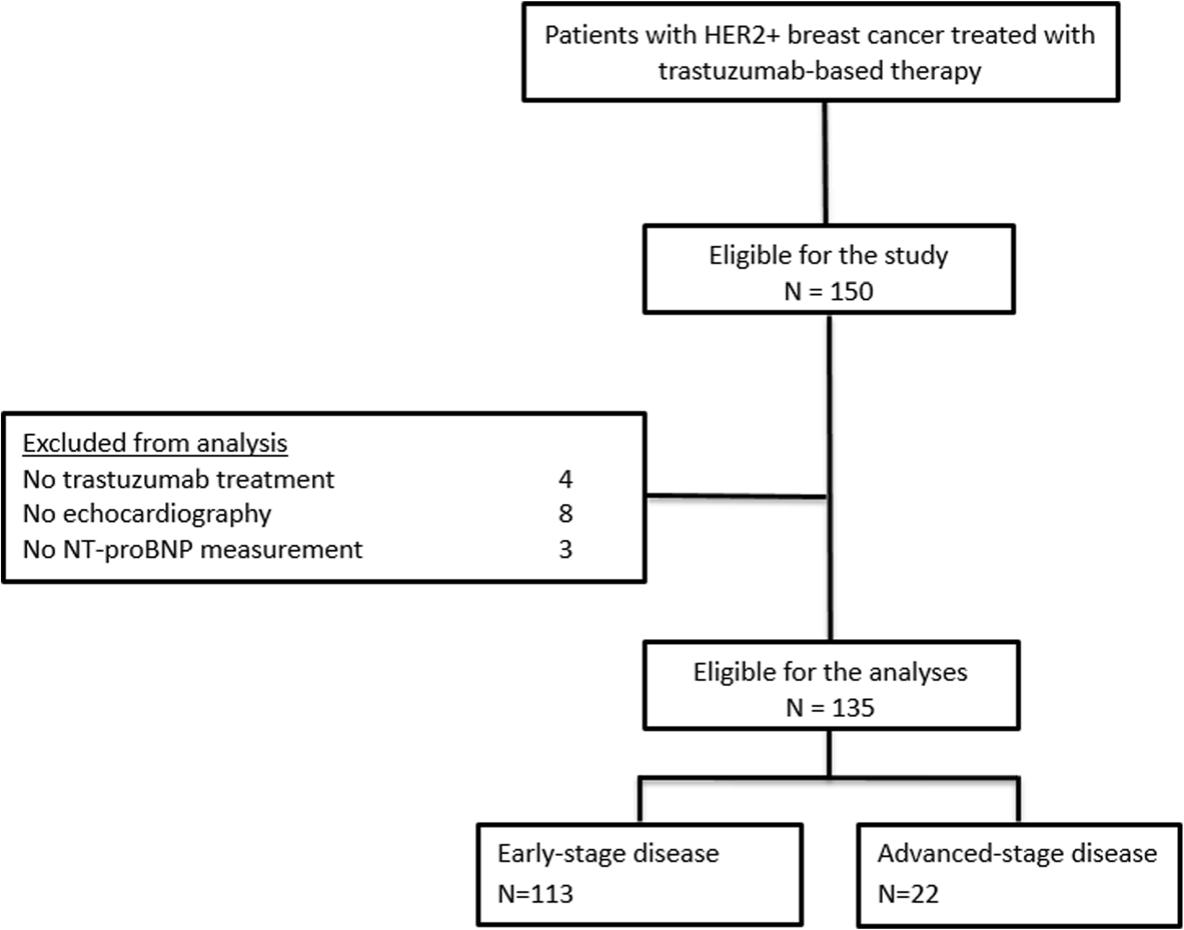
Flowchart of patient inclusion. *Abbreviations: HER2+, Human Epidermal growth factor Receptor positive; NT-proBNP, N-terminal pro-brain natriuretic peptide*

Between April 2008 and June 2016, 150 patients with HER2-positive breast cancer were enrolled. A total of 15 patients were excluded from the analyses because they did not receive trastuzumab treatment (*N* = 4), had no echocardiography (*N* = 8) or NT-proBNP measurement (*N* = 3). Hence, a total of 135 patients were available for analyses (Fig. 1). Median (IQR) age of the patients at inclusion was 54 years (47–61; Table 1). Of all included patients, 113 patients (84%) had early-stage breast cancer, whereas the remaining patients had advanced-stage disease. Prior cardiac disease including valve insufficiency, arrhythmia and myocardial ischemia was present at start of treatment in 10 of the included patients (7%).

**Table 1:** Patient characteristics

Characteristics	Total group (*N* = 135) [IQR], (%)	Early-stage (*N* = 113) [IQR], (%)	Advanced-stage (*N* = 22) [IQR], (%)
Age (years)	54 [47–61]	53 [47–60]	61 [53–65]
BMI (kg/m^2^)	25.6 [23.7–29.5]	25.6 [23.6–29.4]	26.1 [23.9–30.1]
Duration of trastuzumab treatment (months)	11 [11–12]	11 [11–11]	16 [9–44]
Pretreatment with anthracycline (doxorubicin)	111 (82)	107 (95)	4 (18)
Local radiotherapy	61 (45)	53 (47)	8 (36)
Cardiac condition before treatment			
• Valve insufficiency	5 (3.5)	3 (2.6)	2 (9)
• Arrhythmia	5 (3.7)	4 (3.5%)	1 (4.5)
• MI/CABG/PCI	0	0	0
LVEF (%)	62 [58–65]	62 [58–65]^a^	61 [57–66]^b^
NT-proBNP (pmol/l)^c^	9 [5–14]	8 [5–14]	11 [7–18]
Follow-up duration (months)^d^	13 [11–14]	13 [11–14]	11 [9–12]

Abbreviations: *BMI* body mass index, *MI* myocardial infarction, *CABG* coronary artery bypass graft, *PCI* percutaneous coronary intervention, *LVEF* left ventricle ejection fraction, *NT-proBNP* N-terminal pro-brain natriuretic peptide, *IQR* interquartile range

^a^Measured at T0

^b^Measured at T1

^c^Measured at baseline

^d^Calculated from start anthracycline treatment to last available LVEF or NT-proBNP measurement

### Trastuzumab-induced cardiotoxicity

During the 1 year of follow-up, 4 patients (3%) died. All patients died because of disease progression (Table 2). The range of the last available LVEFs of these patients was 53 to 62%.

**Table 2:** Clinical outcomes during 1 year of trastuzumab treatment

Clinical outcomes	Total group (*N* = 135), (%)	Early-stage (*N* = 113), (%)	Advanced-stage (*N* = 22), (%)
Cardiac events			
Trastuzumab-induced cardiotoxicity			
Solely LVEF < 45%	1 (1)	0	1 (5)
Solely absolute LVEF decline > 10% from baseline	29 (21)	26 (23)	3 (14)
LVEF < 45% and absolute decline > 10% from baseline	15 (11)	12 (11)	3 (14)
Total^a^	45 (33)	38 (34)	7 (32)
Atrial fibrillation	0	0	0
Myocardial ischemia	0	0	0
Other cardiac events	0	0	0
Diastolic dysfunction grade 3 or 4	2 (2)	1 (1)	1 (5)
Postponement of trastuzumab			
Temporarily	6 (4)	6 (5)	0
Permanent	14 (10)	12 (11)	2 (9)
Death			
Progression disease	4 (3)	2 (2)	2 (9)

Abbreviations: *3DE* three-dimensional echocardiography, *LVEF* left ventricle ejection fraction; ^a^LVEF < 45% and/or absolute LVEF decline > 10%

In total, 45 patients (33%) developed TIC during treatment with trastuzumab (Table 2), of whom 44 (98%) experienced an absolute LVEF decline of > 10%. A total of 16 (36%) patients displayed an LVEF < 45% during trastuzumab treatment. We found no difference in TIC between patients with early-stage disease and advanced-stage disease (*p* = 0.805). The median time to TIC was 6 months (IQR 5–11 months). Two patients developed diastolic dysfunction of more than grade 2. These patients also demonstrated TIC. No other clinical cardiac events were observed.

The treating physician postponed treatment with trastuzumab in 6 patients temporarily and in 14 patients permanently because of cardiotoxicity. LVEF improved and returned to normal after discontinuation of trastuzumab treatment in all but 2 patients (10%).

### Baseline factors associated with TIC

Age at diagnosis of breast cancer and baseline LVEF were not associated with TIC during 1 year of trastuzumab treatment (Table 3). However, patients who developed TIC showed an LVEF change of −6.6% during anthracycline treatment prior to the start of trastuzumab versus an LVEF change of −0.8% in patients without TIC (*p* = 0.033). The hazard ratio (HR) of an anthracycline-induced LVEF decline for TIC was 1.16. Although patients with TIC did not have significant higher baseline NT-proBNP than patients who remained TIC-free (median of 12 pmol/l versus 8 pmol/l, mean difference of 4 pmol/l, *p* = 0.229), single measurement of NT-proBNP at baseline was related with the development of TIC during follow-up (HR 1.04, 95% CI 1.02–1.07, *p* = 0.003).

**Table 3:** Risk factors associated with TIC during 1 year of trastuzumab treatment

Independent variables	Adjusted HR	95% CI	*P*-value	Patients with TIC^a^	Patients without TIC^a^	*P*-value^b^
NT-proBNP (pmol/l)^c^	1.04	1.02–1.07	0.003	12 [5–19]	8 [5–12]	0.229
LVEF (%)^c^	1.06	0.98–1.15	0.136	64 [60–67]	60 [58–63]	0.003
Absolute LVEF decline (%) during anthracycline treatment	1.16	1.07–1.25	< 0.001	6.6 [2–9]	0.8 [−3–4]	0.033
Age (years)^c^	0.99	0.96–1.02	0.513	53 [47–61]	55 [47–61]	0.296

Abbreviations: *NT-proBNP* N-terminal pro-brain natriuretic peptide, *LVEF* left ventricle ejection fraction, *HR* hazard ratio, *TIC* trastuzumab-induced cardiotoxicity

^a^Median and IQR

^b^*P*-value obtained from comparison of values patients with and without TIC

^c^Measured at baseline

### Temporal evolution of LVEF

A total of 770 3DEs were obtained, of which 9 could not be interpreted because of poor quality, leaving 761 available for analysis, which implies a median of 6 (IQR 5–6) per patient. The median LVEF at baseline was 61% (IQR 59–65%).

**Fig. 2:**
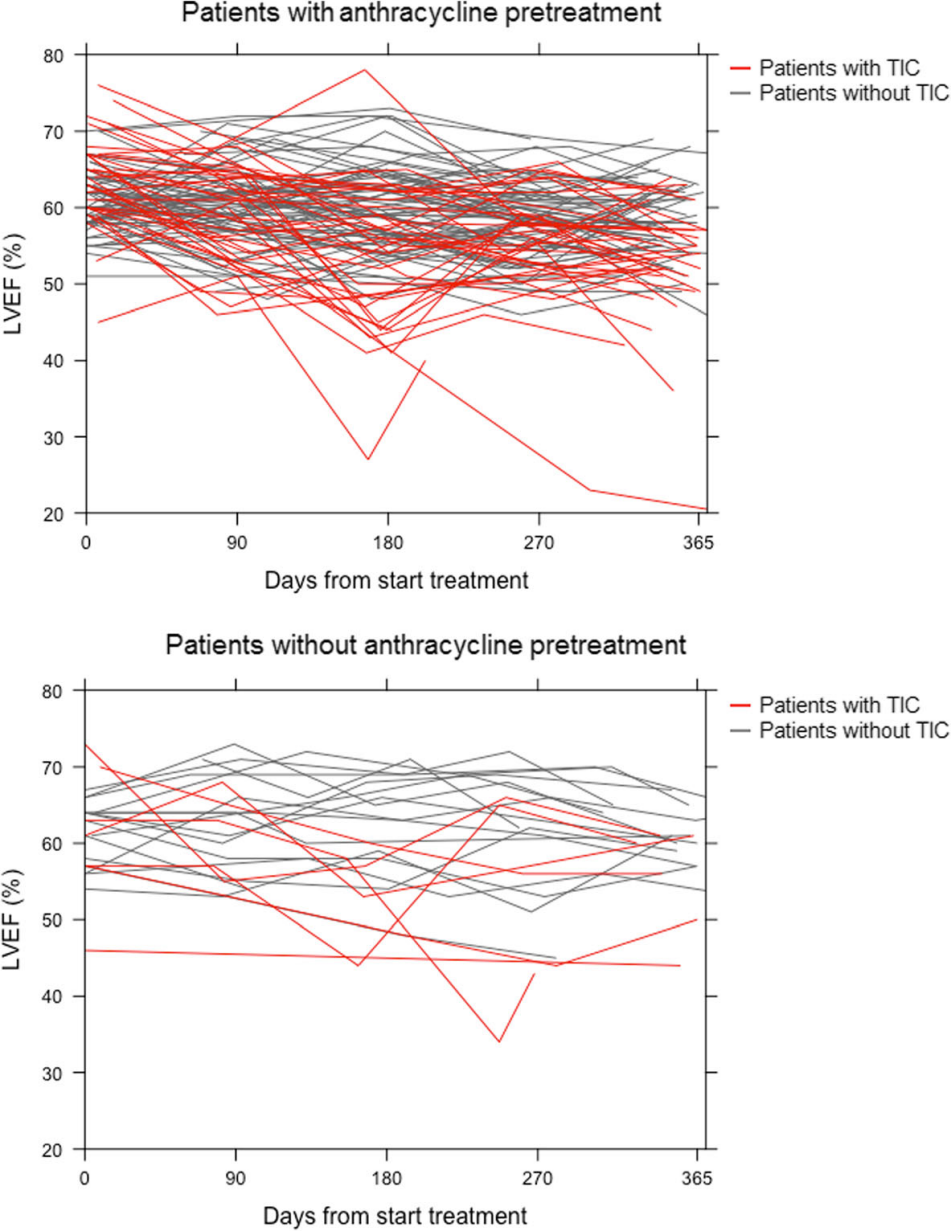
Trajectory of LVEF of patients with TIC and without in patients with and without anthracycline pretreatment. *Abbreviations: LVEF, left ventricle ejection fraction; TIC, trastuzumab-induced cardiotoxicity; NT-proBNP, N-terminal pro-brain natriuretic peptide*

In all patients together, during 1 year of trastuzumab treatment, the mean LVEF declined by 4.5% (95% CI -3.3% to − 5.8%; *p* < 0.001). In fact, this was mainly driven by the patients with TIC, who showed a change of − 9.5% in LVEF (95% CI -7.2% to − 11.7%; *p* = 0.001) as compared to a change of − 1.6% in LVEF (95% CI -0.6% to − 2.7%; *p* = 0.944) in their TIC-free counterparts. A post-hoc analysis of the 107 early-stage disease patients receiving anthracycline treatment demonstrated that, when comparing LVEF before anthracycline treatment  with the LVEF at end of anthracycline treatment, patients with TIC experienced an LVEF decline of 0.056 per day and patients without TIC an LVEF decline of 0.002 per day. Figure 2 shows the trajectory of LVEF of patients with and without TIC in patients with and without anthracycline pretreatment.

### Temporal evolution of NT-proBNP

**Fig. 3:**
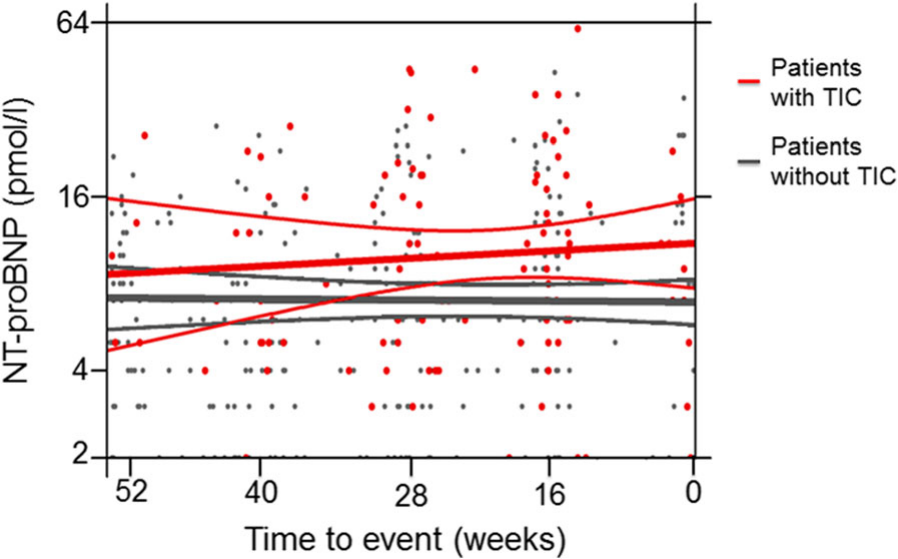
Trajectory of NT-proBNP before TIC or last follow-up of patients without TIC. *Abbreviations: NT-proBNP, N-terminal pro-brain natriuretic peptide; TIC, trastuzumab-induced cardiotoxicity*

A total of 692 NT-proBNP values were determined with a median of 6 per patient (IQR 4–7). NT-proBNP and LVEF were related, and every + 10 pmol/l difference in NT-proBNP (at any time point during follow-up) was associated with an absolute difference in LVEF of − 4.5% (95% CI -2.2% to − 6.7%; *p* < 0.001). Mean levels of NT-proBNP in patients with and without TIC were 16.8 and 10.1 pmol/l, respectively, which implies a mean difference of 6.7 pmol/l (*p* = 0.031). The HR for developing TIC was 1.06 per + 10 pmol/l difference in NT-proBNP at any time point during follow-up (95% CI 1.02–1.10, *p* = 0.008). NT-proBNP levels in all individual patients slightly increased from baseline (+ 2.9 pmol/l), more so in patients with TIC (+ 10.2 pmol/l) than in those without (+ 2.5 pmol/l), and this difference was statistically significant (*p* = 0.037). Interestingly, there was no evidence of a steadily or sudden increase in NT-proBNP prior to TIC. (Fig. 3).

## Discussion

This study in HER2-positive breast cancer patients showed that serum levels of NT-proBNP increased during the first year of trastuzumab treatment. Patients who developed cardiotoxicity showed a steeper increase and had on average higher NT-proBNP levels than those in whom left ventricular function remained preserved. Still, NT-proBNP failed as a biomarker for early identification of cardiac dysfunction, as the changes were too subtle and could barely be distinguished from normal intra-subject variability. [23]

Our observation that NT-proBNP increases during trastuzumab treatment was also described by the studies of Romano et al. and Zardavas et al. [10, 11] Romano et al. also demonstrated that an increase of NT-proBNP during anthracycline is predictive for cardiotoxicity within 3 to 12 months. [10] This is in contrast with various other studies, which neither found increased NT-proBNP values during trastuzumab treatment, nor a relation between NT-proBNP and the incidence of cardiotoxicity. [12–17] The explanation of these variable results is most likely multifactorial, and related to the type of treatment, sample size, the specific NT-proBNP assay used, and, probably most relevant, the definition of cardiotoxicity (Additional file 1: Table S1). In the current study, a clinically relevant and widely accepted international definition of cardiotoxicity was used (absolute LVEF decline of > 10% and/or LVEF < 45%). We opted for this strict definition, as patients will usually only start to experience cardiac symptoms when LVEF levels below 45% are reached. In addition, an absolute LVEF decline of > 10% can suggest an increased risk of heart failure and treatment with an ACE inhibitor is advised. [22] It should also be noted that in the study of Romano et al., patients exclusively received anthracycline-based chemotherapy, whereas trastuzumab-based chemotherapy was not administered.

No evidence was found of a steadily or sudden increase in NT-proBNP before the development of TIC. Therefore, we concluded that NT-proBNP is not suited for the early identification of TIC. Still, in the current study, NT-proBNP actually did increase during trastuzumab treatment, and this increase was indeed related with a declining LVEF. The question on the role of NT-proBNP in detecting cardiotoxicity in patients receiving trastuzumab therefore still remains. The intra- and inter-subject variability of NT-proBNP are noteworthy, and the types of NT-proBNP assays used in the studies differ. To overcome these drawbacks, further research regarding NT-proBNP should be considered in larger, international, multi-center studies.

Whether cardiac biomarkers in general are suitable for detecting cardiotoxicity in patients receiving cardio-toxic cancer treatment also remains questionable. There are multiple pathways that could be involved in the development of cardiotoxicity due to cardio-toxic cancer treatment, and therefore multiple biomarkers may be useful in detecting cardiotoxicity. The pathway of anthracycline involves inhibition of topoisomerase IIb in myocardial cells with apoptosis and radical oxygen species formation as result [24], but less is known about the specific pathway involved in trastuzumab-induced cardiotoxicity. For example, troponin is released in patients with ischemic heart disease, but until now it has not been proven efficient in detecting cardiotoxicity due to trastuzumab treatment. [15–17, 25, 26] More knowledge about the pathway of trastuzumab-induced cardiotoxicity could possibly identify (new) cardiac biomarkers which could be useful for detecting this  cardiotoxicity. Recently, the prognostic value of the biomarker suppressor of tumorgenicity 2 (ST2) became evident for acute [27] and chronic [28] heart failure patients. However, this biomarker, alone or in combinations with other cardiac biomarkers, has not been investigated extensively in patients undergoing cardio-toxic treatment for HER2-positive breast cancer.

Three-dimensional echocardiography, not standard MUGA scan, was used in the current study for the reason that 3DE is the preferred technique for LVEF monitoring and detection of cardiotoxicity due to the high accuracy in detecting LVEF levels below the lower limit of normal, high reproducibility and low temporal variability. [5] Major limitations of measuring LVEF with MUGA scans are the questionable accuracy, the cumulative radiation exposure of serial monitoring, intra-and inter variability and its limited information on other cardiac structures. [5, 7, 29–31] It should be noted that the choice of imaging modality can influence the estimated incidence of cardiotoxicity, and thus the relation with NT-proBNP. The other studies investigating the relationship between NT-proBNP and cardiotoxicity used two-dimensional echocardiography (2DE) for the LVEF assessments. However, 2DE is suggested to overestimate the mean LVEF by 5%. [32] Because 3DE LVEF measurements do not systematically differ from cardiac MRI (CMR) LVEF measurements [32], the gold standard for LVEF measurements, and 3DE has a higher availability than CMR, we used 3DE in this study. However, it cannot be completely excluded that some of our results represent false-positive results.

Interestingly, our study showed that patients with a (steeper than average) decrease in LVEF after anthracycline-based chemotherapy are most likely to develop cardiotoxicity during trastuzumab-based treatment. Thus, importantly, these patients can be identified prior to the start of trastuzumab, as they can potentially benefit from strict cardiac monitoring. This is in line with the European Society for Medical Oncology (ESMO) guidelines that recommend to reassess the LVEF if during anthracycline-based chemotherapy the LVEF declines below the 50% and to consider therapy for left ventricular dysfunction if the LVEF is confirmed to be below the 50% after anthracycline-based chemotherapy. [33] More should be learned about which factors - the cardiac response to anthracycline treatment, or the cardiac response to trastuzumab treatment, or others - are most important regarding the development of cardiotoxicity during trastuzumab treatment.

### Limitations

Several limitations were inherent to the study design and limit the interpretation of our findings. First, all patients were treated and monitored in one single hospital. Although this hospital is a large, secondary, teaching-hospital in the Netherlands, it must be taken into account that external validity of the study findings had not been demonstrated. Secondly, our sample size was possibly too small in regard of the intra-subject variability of both NT-proBNP and LVEF. Although, compared with similar studies on the topic, our study had one of the highest number of included patients (Additional file 1: Table S1). Thirdly, in some cases there was a time delay between the NT-proBNP measurement and 3DE at one time point. Although we only observed a steady and slow increase in NT-proBNP coupled with a steady decrease in LVEF, the timing mismatch might have influenced our findings. At last, the echocardiograms were made by two different persons and reviewed by one cardiologist, so there may be some inter-observer variability in the results of the LVEF. However, 3DE is known to have a low inter-observer variability of 0.027% compared to 2DE. [34]

## Conclusion

NT-proBNP cannot be used as a surrogate monitoring tool for trastuzumab-induced cardiotoxicity in HER2-positive breast cancer patients during the first year of treatment. Patients showing an LVEF decline during anthracycline pretreatment appeared vulnerable for trastuzumab-induced cardiotoxicity.

## Additional file

Additional file 1: **Figure S1.** Study procedures during 1 year follow-up. **Table S1.** Overview of studies investigating the relation of NT-proBNP and cardiotoxicity. (DOCX 63 kb)

## Abbreviations

2DE: Two-dimensional echocardiography
3DE: Three-dimensional echocardiography
ACE: Angiotensin-converting enzyme
BMI: Body mass index
CABG: Coronary artery bypass grafting
CI: Confidence interval
CMR: Cardiac Magnetic Resonance Imaging
DFS: Disease-free survival
EDV: End diastolic volume
ESV: End systolic volume
HER2: Human Epidermal growth factor Receptor 2
HR: Hazard ratio
IQR: Interquartile range
JM: Joint model
LV: Left ventricle
LVEF: Left ventricular ejection fraction
MI: Myocardial infarction
MUGA scan: Multigated acquisition scan
NLME model: Non-linear mixed effects model
NT-proBNP: N-terminal pro-brain natriuretic peptide
OS: Overall survival
PCI: Percutaneous coronary intervention
SD: Standard deviation
ST2: Suppressor of tumorgenicity 2
TIC: Trastuzumab-induced cardiotoxicity
