# Supplementary material for: NT-proBNP correlates with LVEF decline in HER2-positive breast cancer patients treated with trastuzumab
Source: Cardiooncology. 2019 May 28;5:4. doi: 10.1186/s40959-019-0039-4 (PMC7048136; doi:10.1186/s40959-019-0039-4)
Supplement: Supplementary file 1 — Figure S1. Study procedures during 1 year follow-up. Table S1. Overview of studies investigating the relation of NT-proBNP and cardiotoxicity. (DOCX 63 kb) [file 40959_2019_39_MOESM1_ESM.docx]

**Supplemental material**

Figure 1. Study procedures during 1 year follow-up


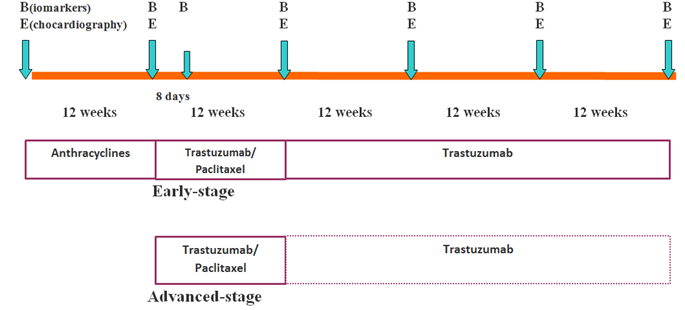


Table 1. Overview of studies investigating the relation of NT-proBNP and cardiotoxicity

| Study | Population | Treatment and duration | Sample size | Definition of cardiotoxicity + imaging modality | Incidence of cardiotoxicity | NT-proBNP changes during trastuzumab treatment | NT-proBNP related to cardiotoxicity | NT-proBNP assays |
| --- | --- | --- | --- | --- | --- | --- | --- | --- |
| Romano et al. (2011) | Breast cancer patients | Anthracycline | 71 | LVEF of ≥20% and/or increase in LVESV of ≥15% from baseline measured with 2DE | NA | + | + | Elecsys |
| Bouwer et al.(2019) | HER2+ breast cancer patients | Anthracycline + trastuzumab for 1 year | 135 | Absolute LVEF decline >10% from baseline and/or LVEF <45% measured with 3DE | 33% | + | - | Dimension Vista 500 |
| Zardavas et al. (2017) | HER2+ breast cancer patients | Anthracycline + trastuzumab for 1 or 2 years | 310 | Asymptomatic absolute LVEF decline >10% from baseline and LVEF <50% | 14% | + | - | Elecsys |
| Putt et al. (2015) | HER2+ breast cancer patients | Anthracycline + trastuzumab for 1 year | 78 | Symptomatic absolute LVEF decline ≥5% to <55% or asymptomatic absolute LVEF decline ≥ 10% to <55% measured with 2DE | 29% | - | - | Dimension Vista 500 |
| Sawaya et al. (2012) | HER2+ breast cancer patients | Anthracycline + taxanes + trastuzumab for 1 year | 81 | Symptomatic absolute LVEF decline ≥5% to <55% or asymptomatic absolute LVEF decline ≥ 10% to <55% measured with 2DE | 32% | - | - | Dimension Vista 500 |
| Fallah-rad et al. (2011) | HER2+ breast cancer patients | Anthracycline + trastuzumab for 1 year | 42 | LVEF decline >10% to <55% from baseline measured with 2DE with symptoms of heart failure | 24% | - | - | Elecsys |
| Ky et al. (2014) | HER2+ breast cancer patients | Anthracycline + taxanes + trastuzumab for 1 year | 78 | Symptomatic absolute LVEF decline ≥5% to <55% or asymptomatic absolute LVEF decline ≥ 10% to <55% measured with 2DE | 24% | - | - | Dimension Vista 500 |
| Ponde et al. (2017) | HER2+ breast cancer patients | Lapatinib + taxanes + trastuzumab for 28 weeks | 280 | Symptomatic congestive heart failure NYHA III or IV measured with 2DE | 4% | - | - | Cobas^®^ |
| Sawaya et al. (2011) | HER2+ breast cancer patients | Anthracycline + trastuzumab | 43 | LVEF decline of ≥5% to <55% with symptoms of heart failure or an asymptomatic LVEF decline ≥10% to <55% measured with 2DE | 21% | - | - | Dimension Vista 500 |

*Abbreviations: NT-proBNP, N-terminal pro-brain natriuretic peptide; LVEF, left ventricle ejection fraction; LVESV, left ventricle end-systolic volume; 2DE, two-dimensional echocardiography; NA, not available; HER2+, Human Epidermal growth factor Receptor 2 positive; 3DE, three-dimensional echocardiography.*
